# Effect of *Euphorbia neriifolia* L. leaf juice on the growth of *Pseudomonas aeruginosa* from otitis externa: an *in vitro* study

**DOI:** 10.3389/fphar.2026.1760885

**Published:** 2026-03-16

**Authors:** Devira Zahara, Muhammad Ichwan, Yohana Simanjuntak

**Affiliations:** 1 Department of Otorhinolaryngology–Head and Neck Surgery, Faculty of Medicine, Universitas Sumatera Utara, Medan, Indonesia; 2 Department of Pharmacology and Therapeutic, Faculty of Medicine, Universitas Sumatera Utara, Medan, Indonesia

**Keywords:** antibacterial activity, *Euphorbia neriifolia*, *in vitro*, MBC, MIC, otitis externa, *Pseudomonas aeruginosa*, sudu-sudu

## Abstract

**Background:**

*Pseudomonas aeruginosa* is the leading bacterial cause of otitis externa and demonstrates increasing resistance to commonly used topical antibiotics. The growing burden of antimicrobial resistance has prompted exploration of plant-derived antibacterial agents. *Euphorbia neriifolia* has been traditionally used for ear-related conditions; however, its activity against *Pseudomonas aeruginosa* from otitis externa remains insufficiently investigated.

**Objective:**

To evaluate the *in vitro* antibacterial activity of fresh *E. neriifolia* leaf juice against *P. aeruginosa* ATCC 27853 and clinical isolates from otitis externa.

**Methods:**

An *in vitro* experimental study was conducted using broth microdilution and disk diffusion assays. Fresh leaf juice was prepared and tested at concentrations ranging from 10% to 100%. Minimum inhibitory concentration (MIC) and minimum bactericidal concentration (MBC) were determined. Disk diffusion was performed at selected concentrations. Phytochemical constituents were analyzed using LC–MS.

**Results:**

LC–MS identified multiple bioactive compounds, including quercetin, chlorogenic acid, kaempferol, gallic acid, and 7-hydroxycoumarin. MIC and MBC values for ATCC 27853 and clinical isolate 1 were 50% and 60%, respectively, while clinical isolate 2 demonstrated MIC at 60% and MBC at 70%. Disk diffusion showed dose-dependent inhibition, with inhibition zones exceeding 20 mm at 60% for ATCC 27853 and isolate 1, and at 70% for isolate 2. Ofloxacin produced larger inhibition zones than the extract across all strains.

**Conclusion:**

*E. neriifolia* leaf juice exhibits significant *in vitro* antibacterial activity against *P. aeruginosa*, including clinical isolates from otitis externa. Although less potent than ofloxacin, its bioactive profile supports further investigation as a potential adjunctive topical therapy.

## Introdcution

Otitis externa is an inflammatory disorder of the external auditory canal and represents one of the most common conditions encountered in otorhinolaryngology practice worldwide. Its incidence is particularly high in tropical climates, where increased humidity and temperature compromise the epithelial barrier and promote microbial proliferation (Wiegand et al., 2019). Clinically, patients present with otalgia, pruritus, and otorrhea, symptoms that may significantly impair quality of life (Jackson and Geer, 2023). Repeated exposure to contaminated water further increases susceptibility, leading to the well-known term “swimmer’s ear” (Wiegand et al., 2019). In Indonesia, *Pseudomonas aeruginosa* was identified in 42.4% of otitis externa cultures in a tertiary hospital study (Heward et al., 2018).

More than 90% of otitis externa cases are bacterial in origin, with *P. aeruginosa* and *Staphylococcus* aureus as the predominant pathogens (Wiegand et al., 2019). Among these pathogens, *P. aeruginosa* poses a significant therapeutic challenge due to its intrinsic and acquired resistance mechanisms, including efflux pump overexpression, reduced outer membrane permeability, and remarkable capacity for biofilm formation. Biofilm formation allows *P. aeruginosa* to adhere to epithelial surfaces and produce an extracellular polymeric matrix composed of polysaccharides, proteins, and extracellular DNA (Moradali et al., 2017). This matrix acts as a physical and biochemical barrier that limits antibiotic penetration, enhances horizontal gene transfer, and protects bacterial cells from host immune responses. As a result, biofilm-associated infections are often persistent and significantly more resistant to antimicrobial therapy compared with planktonic bacteria (Murray et al., 2022; Langendonk et al., 2021). The global rise in antimicrobial resistance (AMR) has further complicated treatment strategies, with increasing reports of multidrug-resistant strains. In response, the World Health Organization has classified resistant *P. aeruginosa* as a critical priority pathogen requiring urgent development of novel antimicrobial agents (Lyu et al., 2023). Increasing resistance to commonly used topical antibiotics in otitis externa has also been documented (Mali and Panchal, 2017; Sultana et al., 2022).

The growing threat of AMR has renewed interest in plant-derived antimicrobials as potential alternative or adjunctive therapies. Medicinal plants synthesize diverse secondary metabolites—such as flavonoids, phenolic acids, terpenoids, and alkaloids—that exhibit antibacterial, anti-inflammatory, and antioxidant properties (Cushnie and Lamb, 2011; Paczkowski et al., 2016). These compounds constitute a valuable source for the discovery of novel antibacterials, particularly against pathogens exhibiting increasing resistance (Paczkowski et al., 2016).

*Euphorbia neriifolia L.,* locally known as sudu-sudu, is widely distributed across South and Southeast Asia and has long been used in traditional medicine for various inflammatory and infectious conditions (Lou et al., 2011; Borges et al., 2013; Nowakowska, 2007). Phytochemical investigations have identified bioactive constituents such as euphol, taraxerol, β-amyrin, nerifoliol, and other flavonoid and phenolic compounds with reported antimicrobial and anti-inflammatory effects (Nowakowska, 2007; Venugopala et al., 2013). Ethnomedicinal reports describe the use of fresh leaf juice for ear-related complaints, suggesting potential ototopical applications (Borges et al., 2013). Previous *in vitro* studies have demonstrated antibacterial activity of *Euphorbia neriifolia L.* against several bacterial species using diffusion-based assays (Costerton et al., 1999).

Despite these promising findings, the antibacterial activity of fresh *E. neriifolia* leaf juice against *P. aeruginosa*, particularly clinical isolates derived from otitis externa, has not been systematically evaluated. This represents an important knowledge gap in the context of rising antimicrobial resistance and the need for safe, locally accessible adjunctive therapies.

Therefore, this study aimed to evaluate the *in vitro* antibacterial activity of fresh *E. neriifolia* leaf juice against *P. aeruginosa* ATCC 27853 and clinical isolates obtained from otitis externa by determining the minimum inhibitory concentration (MIC), minimum bactericidal concentration (MBC), and inhibition zone diameters using standardized susceptibility assays.

## Methods

### Study design

This study was an *in vitro* laboratory experimental study employing a post-test only control group design. The experimental setup consisted of one positive control group treated with ofloxacin (5 μg), one negative control group treated with sterile aquabides, and ten treatment groups exposed to *E. neriifolia* fresh leaf juice at concentrations of 10%, 20%, 30%, 40%, 50%, 60%, 70%, 80%, 90%, and 100%. The concentration range (10%–100%) was selected to establish a dose–response relationship and to enable identification of the minimum inhibitory concentration (MIC) and minimum bactericidal concentration (MBC). The highest concentration (100%) represented the undiluted crude fresh leaf juice, while graded 10% incremental dilutions allowed systematic and biologically relevant evaluation of antibacterial activity across increasing concentration levels. Antibacterial activity against *P*. *aeruginosa* ATCC 27853 (American Type Culture Collection [ATCC], Manassas, VA, United States) and two clinical isolates obtained from the Microbiology Laboratory of Sumatera Utara, Indonesia, was assessed using microdilution and disk diffusion assays. This design allowed direct comparison of bacterial growth inhibition and bactericidal effects across all treatment concentrations and control groups.

### Study setting and timeline

The study was conducted in July 2025 at several facilities. Ear discharge samples from otitis externa patients were collected at the ENT Outpatient Clinic of Prof. Chairuddin P. Lubis Hospital, Medan. Botanical identification of *E. neriifolia L*. was performed by Prof. Dr. Etti Sartina Siregar, S.Si., M.Si., a taxonomist at the Faculty of Mathematics and Natural Sciences, Universitas Sumatera Utara (USU). Preparation of the fresh leaf juice was carried out in the Pharmacology Laboratory, Faculty of Medicine, USU. Bacterial culture, inoculum preparation, and antibacterial testing—including microdilution and disk diffusion assays—were conducted.

### Study population and samples

The study population consisted of 2 patients diagnosed with otitis externa based on clinical symptoms and otoscopic examination. The accessible population included patients with confirmed *P. aeruginosa* infection presenting to the ENT Outpatient Clinic in August 2025. Sample selection followed a non-probability consecutive sampling technique.

### Sample size calculation

Sample size was determined using Federer’s formula for experimental designs, $\left(t–1\right)\left(n–1\right)\geq 15$ , where *t* denotes the number of treatment groups and *n* the number of replications per group (Federer, 1967). In this study, a total of 12 experimental groups were included (10 extract concentrations and 2 control groups). Substituting into the formula yielded $\left(12–1\right)\left(n–1\right)\geq 15$ , resulting in *n* ≥ 2.36. Therefore, each experimental condition was performed in triplicate. This resulted in a minimum total of 36 experimental units to ensure adequate statistical reliability.

### Inclusion and exclusion criteria

Clinical isolates were included if they were confirmed as *P. aeruginosa* using the Vitek® 2 Compact automated identification system (bioMérieux, France), based on biochemical profiling and automated species-level identification. Antimicrobial susceptibility testing was performed using the same system, and interpretation of ofloxacin susceptibility was based on the Clinical and Laboratory Standards Institute (CLSI) breakpoints (CLSI M100, current edition at the time of testing). Isolates were categorized as susceptible if the minimum inhibitory concentration (MIC) values met CLSI-defined susceptibility thresholds. A total of two clinical isolates were initially collected from two patients diagnosed with otitis externa. All collected isolates were confirmed as *P. aeruginosa* and were susceptible to ofloxacin; therefore, no isolates were excluded due to antimicrobial resistance. Samples were considered “damaged” if there was leakage of transport medium, desiccation of the swab, contamination with visible mixed bacterial growth inconsistent with *P. aeruginosa*, or failure to yield viable growth upon subculture. No samples met these exclusion criteria.

## Materials and equipment

Standard microbiological laboratory equipment was used, including a biosafety cabinet, autoclave, analytical balance, microplates, vortex mixer, colony counter, micropipettes, incubator, hotplate, Petri dishes, cotton swabs, cooler box, spiritus burner, sterile test tubes, Erlenmeyer flasks, and other sterile consumables. Bacterial identification and antibiotic susceptibility testing were performed using the Vitek 2 Compact system. Mueller–Hinton Agar (MHA) and Mueller–Hinton Broth (MHB) (Oxoid Ltd., Basingstoke, United Kingdom) were used as the primary culture media for all antibacterial assays, sterile aquabides, and fresh *E. neriifolia L.* leaves were used as primary materials.

### Preparation of *Euphorbia neriifolia* leaf juice

Fresh, mature leaves of *E. neriifolia L.* were collected in July 2025 from cultivated plants in Medan, North Sumatra, Indonesia. Only fully expanded, disease-free leaves at the vegetative stage were selected. A total of 45 fresh leaves were harvested, yielding 930 g of fresh material. Botanical authentication was performed by a certified botanist at the Faculty of Mathematics and Natural Sciences, Universitas Sumatera Utara, and a voucher specimen (No. EN-07-2025) was deposited in the Herbarium Medanense for future reference. The leaves were washed under running distilled water to remove debris and surface contaminants and gently blotted dry with sterile gauze. Initial shade-drying was performed in a well-ventilated area at ambient temperature (27 °C–30 °C) for 24 h to reduce surface moisture while minimizing degradation of thermolabile phytochemicals. The fresh weight prior to drying was 930 g, and the post-drying weight was 840 g. Subsequently, the leaves were subjected to mild heat treatment in a hot-air oven at 80 °C for 5–10 min to soften plant tissues and facilitate mechanical pressing. This amount (930 g) represented the total harvested biomass available during the collection period and was processed entirely to ensure homogeneity of the extract. The softened leaves (840 g) were mechanically pressed using a sterile stainless-steel press extractor. The crude juice was filtered sequentially through sterile muslin cloth and Whatman No. 1 filter paper to remove particulate matter. A total volume of 268 mL of clear green crude juice was obtained, corresponding to an extraction yield of approximately 31.9% (v/w). The crude juice was considered as 100% concentration. Serial dilutions (10%–90%) were prepared using sterile distilled water (aquabides) under aseptic conditions. All extracts were freshly prepared on the day of antibacterial testing and stored at 4 °C for no longer than 24 h prior to use. Qualitative phytochemical screening revealed the presence of flavonoids, phenolic compounds, tannins, saponins, and terpenoids. Further chemical characterization was performed using Liquid Chromatography–Mass Spectrometry (LC–MS) at PT Corpora Science. Separation was achieved using a C18 reversed-phase column (150 mm × 4.6 mm, 5 µm particle size). The mobile phase consisted of solvent A (0.1% formic acid in water) and solvent B (acetonitrile), applied in a gradient elution mode at a flow rate of 0.5 mL/min. The injection volume was 10 μL, and detection was performed using electrospray ionization (ESI) in both positive and negative ion modes. Mass spectra were acquired over an m/z range of 100–1000. Tentative identification of secondary metabolites was performed by comparing mass fragmentation patterns with available spectral libraries and literature data.

### Preparation of bacterial inoculum

Bacterial inocula were prepared using the direct colony suspension method. Well-isolated colonies of *P. aeruginosa* ATCC 27853 and the two clinical isolates were transferred into sterile distilled water (aquabides) and vortexed until a homogeneous suspension was obtained. The turbidity of each suspension was adjusted to match the 0.5 McFarland standard using a calibrated nephelometer (bioMérieux, France). Calibration of the instrument was verified using a commercial McFarland turbidity standard (0.5 McFarland equivalent to approximately 1.5 × 10^8^ CFU/mL) according to the manufacturer’s instructions prior to inoculum preparation. The standardized suspension was subsequently diluted in Mueller–Hinton Broth to obtain a final working inoculum of approximately 1 × 10^6^ CFU/mL for antibacterial assays.

### Broth microdilution assay for MIC and MBC determination

Minimum inhibitory concentration (MIC) and minimum bactericidal concentration (MBC) were determined using the broth microdilution method in accordance with Clinical and Laboratory Standards Institute (CLSI) guidelines with minor modifications. Briefly, a 96-well sterile flat-bottom microtiter plate was used. Each well contained a final volume of 200 μL consisting of 100 μL Mueller–Hinton Broth (MHB) and 100 μL of *E. neriifolia* leaf juice at the designated concentration (10%–100%). A standardized bacterial suspension adjusted to 0.5 McFarland standard (approximately 1 × 10^8^ CFU/mL) was further diluted 1:100 in MHB to achieve a final inoculum concentration of approximately 1 × 10^6^ CFU/mL. Subsequently, 10 μL of this suspension was added to each well, resulting in a final bacterial density of approximately 5 × 10^5^ CFU/mL per well.

The following controls were included:

- Positive control: ofloxacin (5 μg/mL) with bacterial inoculum
- Growth control: MHB with bacterial inoculum (no extract)
- Negative control (sterility control): MHB only (no bacteria, no extract)

Plates were incubated at 37 °C for 18–24 h under aerobic conditions.

The MIC was defined as the lowest concentration of extract showing no visible turbidity compared to the growth control. For MBC determination, 10 μL from wells showing no visible growth were subcultured onto Mueller–Hinton Agar plates and incubated at 37 °C for 24 h. The MBC was defined as the lowest concentration showing no colony growth on agar plates, indicating ≥99.9% bacterial killing. All experiments were performed in triplicate on three independent occasions.

### Disk diffusion assay

The antibacterial activity of *E. neriifolia* leaf juice was further evaluated using the disk diffusion method in accordance with CLSI guidelines. A standardized bacterial suspension equivalent to 0.5 McFarland standard (approximately 1 × 10^8^ CFU/mL) was prepared and uniformly inoculated onto Mueller–Hinton Agar (MHA) plates using sterile cotton swabs to create a confluent lawn culture. The agar depth was maintained at 4 mm as recommended by CLSI. Sterile blank filter-paper discs (6 mm diameter) were impregnated with 20 μL of leaf juice at selected concentrations based on MIC and MBC findings. The discs were allowed to dry under aseptic conditions before placement on the inoculated agar surface. Ofloxacin (5 μg) commercial discs were used as the positive control. Discs impregnated with sterile distilled water (aquabides) served as the negative control. All plates were incubated aerobically at 37 °C for 18–24 h. After incubation, inhibition zone diameters were measured in millimeters using a calibrated digital caliper. Measurements were taken in two perpendicular directions, and the mean value was recorded. All experiments were conducted in triplicate for each bacterial strain.

### Data processing and statistical analysis

All numerical data were tabulated and analyzed statistically. Data normality was assessed using the Shapiro–Wilk test. Normally distributed data were analyzed using one-way ANOVA with a significance level of p < 0.05, while non-normally distributed data were analyzed using the Kruskal–Wallis test. Post hoc analyses were conducted to evaluate differences in inhibition zone diameters between treatment groups. Effect size was calculated using Cohen’s *d* to assess the magnitude of antibacterial activity of *E. neriifolia* leaf juice relative to control groups.

## Results

### LC–MS analysis of *Euphorbia neriifolia* leaf juice extract

LC–MS analysis identified several secondary metabolites in the fresh *E. neriifolia* leaf juice, including flavonoids, phenolic acids, organic acids, and coumarin derivatives (Table 1). Quercetin, a flavonol compound, has been reported to exert antibacterial activity against Gram-negative bacteria through disruption of cell membrane permeability and inhibition of nucleic acid synthesis (CLSI, 2023). Kaempferol has demonstrated inhibitory effects against *P. aeruginosa* by interfering with quorum sensing and biofilm formation (CLSI, 2023). Chlorogenic acid has been shown to increase membrane permeability and induce cytoplasmic leakage in Gram-negative bacteria (Nostro and Papalia, 2012). Gallic acid exhibits bactericidal activity via oxidative stress induction and membrane destabilization (Nostro and Papalia, 2012). Sulfurein, a chalcone derivative, has been reported to possess antibacterial and anti-inflammatory properties through modulation of bacterial enzymatic pathways (Nostro and Papalia, 2012). Additionally, 7-hydroxycoumarin (umbelliferone) has demonstrated antimicrobial effects attributed to inhibition of bacterial DNA gyrase and suppression of biofilm development (Nostro and Papalia, 2012). The presence of these bioactive compounds provides mechanistic support for the antibacterial activity observed against *P. aeruginosa* in this study.

**TABLE 1:** LC-MS profile of metabolites detected in *Euphorbia neriifolia* leaf juice.

No	Compound	Biological activity
1	2-Oxoglutaric acid	Organic acid: lowers pH
2	Fumaric acid	Organic acid: lowers pH
3	Sulfurein	Antibacterial, antioxidant, anti-inflammatory
4	Chlorogenic acid	Polyphenol: antioxidant, antibacterial, anti-inflammatory
5	Kaempferol	Flavonoid: antioxidant, anti-inflammatory, antibacterial
6	5-(5,7-Dihydroxy-3-methoxy-4-oxo-4H-chromen-2-yl)-2-hydroxyphenyl β-D-xylopyranoside	Flavonoid: antioxidant, anti-inflammatory, antibacterial
7	7-Hydroxycoumarin	Coumarin: antioxidant, anti-inflammatory, antibacterial
8	Aesculin (Esculin)	Coumarin: antioxidant, anti-inflammatory, antibacterial
9	1,3,5-trihydroxy-4-{[(2E)-3-(3-hydroxy-4-methoxyphenyl)prop-2-enoyl]oxy}cyclohexane-1-carboxylic acid	Antioxidant, anti-inflammatory, antibacterial
10	Quercetin	Flavonoid: antioxidant, anti-inflammatory, antibacterial
11	NP-000587	Flavonoid: antioxidant, anti-inflammatory
12	3-Hydroxy-5-oxo-2,3-oxepanedicarboxylic acid	Antioxidant, antibacterial
13	Gallic acid	Antibacterial, anti-inflammatory, antioxidant
14	Nicotinic acid (Niacin)	Anti-inflammatory
15	Ferulic acid	Antibacterial, anti-inflammatory, antioxidant

^a^ Compounds were tentatively identified based on LC–MS, spectral library matching; confirmation with authentic standards was not performed.

^b^ The analysis was qualitative; retention times and quantitative concentrations were not determined.

^c^ Reported biological activities are based on previously published literature.

^d^ NP-000587 denotes a tentative library match requiring further structural confirmation.

### Identification and antibiotic susceptibility of *Pseudomonas aeruginosa*

Identification and antibiotic susceptibility testing were performed using the Vitek 2 Compact system. The ATCC 27853 strain demonstrated broad susceptibility to antipseudomonal agents, while both clinical isolates exhibited resistance to several β-lactam antibiotics. Clinical Isolate 2 showed slightly higher resistance characteristics compared to Clinical Isolate 1, as reflected by its higher meropenem MIC value.

### Broth microdilution assay (MIC and MBC determination)

The antibacterial activity of *E. neriifolia* leaf juice demonstrated a concentration-dependent inhibitory effect against all tested strains. For *P. aeruginosa* ATCC 27853, bacterial growth remained >300 CFU/mL at concentrations between 10% and 40%. A marked reduction in colony count was observed at 50% (mean 105 CFU/mL), while complete inhibition (0 CFU/mL) occurred at ≥60%, establishing MIC at 50% and MBC at 60% (Table 2).

**TABLE 2:** Colony counts of *Pseudomonas aeruginosa* ATCC 27853 after exposure to leaf juice.

Group	Rep I	Rep II	Rep III	Mean
PDS 10%	>300	>300	>300	>300
PDS 20%	>300	>300	>300	>300
PDS 30%	>300	>300	>300	>300
PDS 40%	>300	>300	>300	>300
PDS 50%	100	108	106	105
PDS 60%	0	0	0	0
PDS 70%	0	0	0	0
PDS 80%	0	0	0	0
PDS 90%	0	0	0	0
PDS 100%	0	0	0	0
Positive control	0	0	0	0
Negative control	>300	>300	>300	>300

### Antibacterial activity of *Euphorbia neriifolia* fresh leaf juice against *Pseudomonas aeruginosa* clinical isolate 1

Clinical Isolate 1 demonstrated a similar response pattern. Growth persisted at concentrations up to 40%, with a substantial reduction at 50% (mean 128 CFU/mL). Complete inhibition was observed at ≥60%, indicating MIC at 50% and MBC at 60% (Table 3).

**TABLE 3:** Colony counts of Clinical Isolate 1.

Group	Rep I	Rep II	Rep III	Mean
PDS 10%	>300	>300	>300	>300
PDS 20%	>300	>300	>300	>300
PDS 30%	>300	>300	>300	>300
PDS 40%	>300	>300	>300	>300
PDS 50%	119	122	143	128
PDS 60%–100%	0	0	0	0
Positive control	0	0	0	0
Negative control	>300	>300	>300	>300

### Antibacterial activity of *Euphorbia neriifolia* fresh leaf juice against *Pseudomonas aeruginosa* clinical isolate 2

In contrast, Clinical Isolate 2 required higher concentrations for bactericidal activity. Growth remained >300 CFU/mL up to 50%, with only partial reduction at 60% (mean 283 CFU/mL). Complete inhibition occurred at ≥70%, establishing MIC at 60% and MBC at 70% (Table 4).

**TABLE 4:** Colony counts of Clinical Isolate 2.

Group	Rep I	Rep II	Rep III	Mean
PDS 10%–50%	>300	>300	>300	>300
PDS 60%	280	277	292	283
PDS 70%–100%	0	0	0	0
Positive control	0	0	0	0
Negative control	>300	>300	>300	>300

These findings indicate variable susceptibility among strains, with Clinical Isolate 2 demonstrating relatively greater resistance.

### Disc diffusion inhibition zones

**FIGURE 1:**
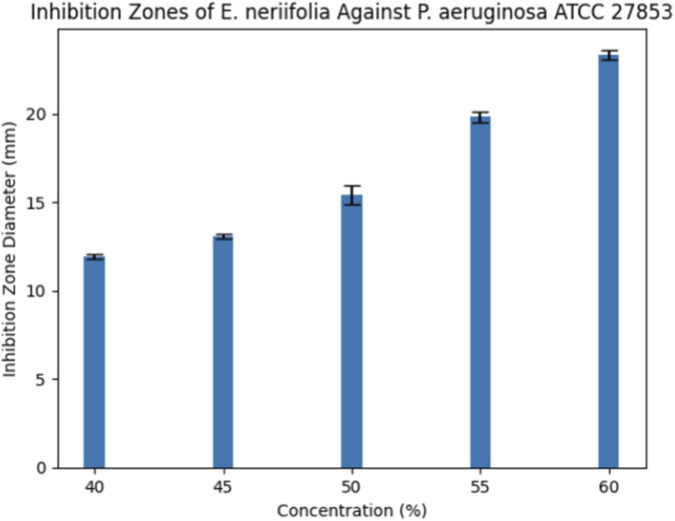
Bar graph inhibition zones ATCC.

A clear dose-dependent increase in inhibition zone diameter was observed across increasing concentrations of *E. neriifolia* leaf juice for all bacterial strains. For ATCC 27853, inhibition zones increased progressively from 11.92 ± 0.14 mm at 40% to 23.33 ± 0.29 mm at 60% (Figure 1). The positive control (ofloxacin 5 μg) produced a significantly larger inhibition zone (35.67 ± 0.76 mm), while the negative control showed no inhibitory activity.

**FIGURE 2:**
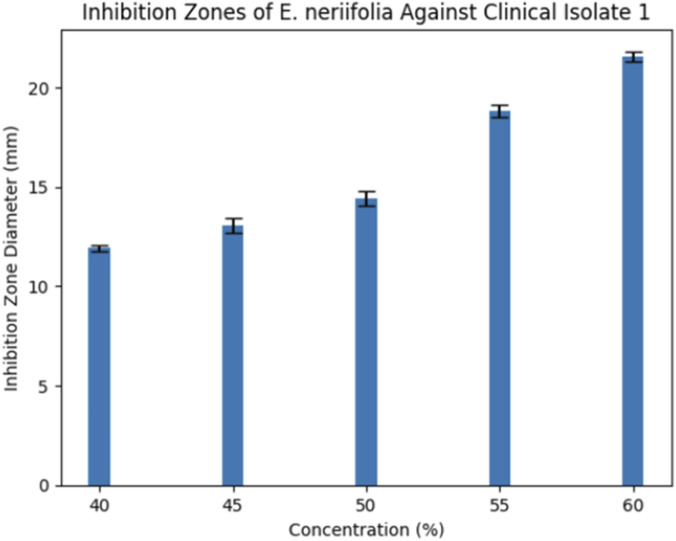
Bar graph inhibition zones Isolate 1.

Clinical Isolate 1 demonstrated a similar concentration-dependent pattern, with inhibition zones increasing from 11.92 ± 0.14 mm at 40% to 21.57 ± 0.25 mm at 60% (Figure 2). Although antibacterial activity was substantial, it remained lower than that of ofloxacin.

**FIGURE 3:**
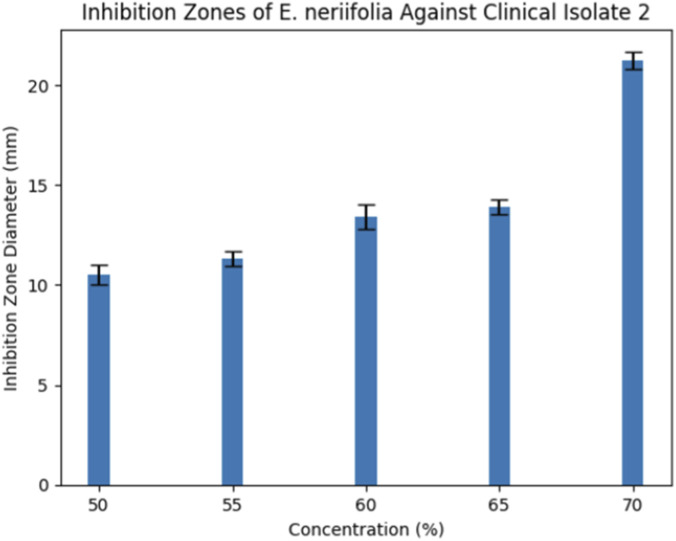
Bar graph inhibition zones Isolate 2.

Clinical Isolate 2 required higher concentrations to achieve comparable inhibition. A pronounced increase in inhibition zone diameter was observed at 70% (21.25 ± 0.43 mm), consistent with its higher MIC and MBC values (Figure 3).

Statistical analysis demonstrated significant differences among treatment concentrations (p < 0.05). Effect size analysis revealed very large antibacterial effects compared with the negative control, confirming the biological relevance of the extract’s inhibitory activity (Table 5).

**TABLE 5:** Summary of statistical analysis of inhibition zones.

Bacterial strain	Overall p-value	Cohen’s d vs. negative control (range)	Effect size interpretation
ATCC 27853	0.003	29.65–93.43	Extremely large
Clinical isolate 1	<0.05	34.42–86.28	Extremely large
Clinical isolate 2	<0.05	21.00–49.42	Extremely large

**FIGURE 4:**
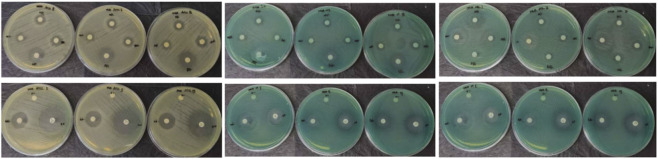
Representative disk diffusion images (ATCC 27853; Isolate 1; Isolate 2).

Representative disk diffusion images are presented in Figure 4.

## Discussion

This study evaluated the *in vitro* antibacterial activity of fresh *E. neriifolia* leaf juice against *P. aeruginosa* ATCC 27853 and two clinical isolates obtained from patients with otitis externa. Compared with the negative control (sterile aquabides), the extract demonstrated a concentration-dependent reduction in bacterial growth, as evidenced by decreased colony-forming units in the broth microdilution assay and increased inhibition zones in the disk diffusion test. However, the antibacterial effect remained lower than that observed with the positive control (ofloxacin 5 µg). These findings indicate preliminary antibacterial potential of the fresh leaf juice and are consistent with previous reports describing antimicrobial activity of phenolic and flavonoid compounds present in *E. neriifolia* and related species (Nostro and Papalia, 2012; Vikram et al., 2010; Breidenstein et al., 2011).

### Phytochemical profile and antibacterial relevance

LC–MS qualitative profiling identified several phenolic and flavonoid compounds, including quercetin, kaempferol, chlorogenic acid, gallic acid, sulfurein, and 7-hydroxycoumarin. Among these, quercetin has been reported to exhibit antibacterial activity against *P. aeruginosa* through disruption of membrane permeability and inhibition of DNA gyrase, with reported MIC values ranging from 125 to 500 μg/mL *in vitro* (Flemming and Wingender, 2010). Kaempferol has demonstrated inhibition of quorum sensing and biofilm formation in *P. aeruginosa*, thereby reducing virulence factor production (Breidenstein et al., 2011). Chlorogenic acid has been shown to increase outer membrane permeability in Gram-negative bacteria and induce cytoplasmic leakage (Vikram et al., 2010), while gallic acid exerts bactericidal effects via oxidative stress induction and membrane destabilization (CLSI, 2023).

In the present study, complete growth inhibition of *P. aeruginosa* isolates was observed at extract concentrations ≥60%, suggesting that the combined presence of these phenolic and flavonoid compounds may contribute to a synergistic antibacterial effect. Although the extract’s MIC was higher compared to standard antibiotics such as ofloxacin, the observed concentration-dependent inhibition supports the biological plausibility of these metabolites contributing to antibacterial activity. Previous studies on *Euphorbia* species have similarly attributed antimicrobial effects to flavonoid and phenolic constituents (Nostro and Papalia, 2012).

### Activity against reference and clinical strains

The leaf juice exhibited consistent antibacterial activity against both the reference strain and clinical isolates. For ATCC 27853 and Clinical Isolate 1, MIC and MBC values were 50% and 60%, respectively. Clinical Isolate 2 required higher concentrations (MIC 60%, MBC 70%), suggesting relatively greater resistance. This variation is consistent with the known heterogeneity of *P. aeruginosa* resistance mechanisms, including biofilm formation and reduced membrane permeability (Breidenstein et al., 2011). The higher concentrations required for complete inhibition in Clinical Isolate 2 may reflect adaptive resistance mechanisms commonly observed in clinical settings. Importantly, the extract retained bactericidal activity even against strains exhibiting partial resistance to conventional β-lactam antibiotics. Although *P. aeruginosa* is well known for its biofilm-forming capacity, particularly in chronic otitis externa, the present study focused primarily on evaluating antibacterial activity against planktonic bacterial growth using standard MIC and MBC assays. Anti-biofilm activity was not assessed because the study was designed as an initial *in vitro* screening to determine growth inhibition and bactericidal effects. Biofilm-specific assays, such as crystal violet quantification or confocal microscopy analysis, were beyond the scope of the current experimental setup. Future studies should investigate the potential anti-biofilm effects of *E. neriifolia* leaf juice, particularly given that flavonoids such as quercetin and kaempferol have been reported to inhibit quorum sensing and biofilm formation in *P. aeruginosa*. Evaluating these effects would provide a more comprehensive understanding of its therapeutic potential in biofilm-associated infections.

### Disk diffusion findings and statistical significance

Disk diffusion results demonstrated a clear dose–response relationship, with inhibition zones increasing proportionally to extract concentration. Inhibition zones exceeded 20 mm at higher concentrations for all strains, indicating substantial antibacterial activity. Although ofloxacin produced larger inhibition zones, the extract exhibited statistically significant differences across treatment concentrations (p < 0.05), with very large effect sizes compared to the negative control. These findings confirm that the observed antibacterial activity is biologically meaningful rather than attributable to random variation. The strong effect sizes reflect the marked contrast between treated groups and the absence of inhibition in the negative control.

### Clinical implications

The findings are particularly relevant in the context of otitis externa, where *P. aeruginosa* remains the predominant pathogen. Beyond antibacterial effects, the presence of anti-inflammatory and antioxidant metabolites may offer additional therapeutic benefits when applied topically. While the extract demonstrated lower potency compared with ofloxacin, its multimodal phytochemical profile suggests potential as an adjunctive or complementary therapy, particularly in settings with limited antibiotic availability or rising antimicrobial resistance (Vikram et al., 2010; CLSI, 2023).

### Study limitations

Several limitations should be acknowledged. First, the study was conducted entirely *in vitro*, and clinical efficacy cannot be directly inferred. Second, the extract used was crude fresh juice without fractionation or standardization of active compound concentrations. Variability in phytochemical composition may influence reproducibility. Third, only two clinical isolates were evaluated, limiting generalizability. Future studies should investigate purified fractions, evaluate antibiofilm activity, and explore *in vivo* or clinical applications to determine safety, optimal dosing, and therapeutic efficacy. Quantitative analysis of individual metabolites was not performed; therefore, the contribution of each compound to antibacterial activity could not be precisely determined.

## Conclusion

This study provides preliminary *in vitro* evidence that fresh *E. neriifolia* leaf juice exerts antibacterial activity against *P. aeruginosa*, including clinical isolates associated with otitis externa. The extract demonstrated concentration-dependent growth inhibition, although its antibacterial potency was lower than that of standard antibiotic therapy. These findings suggest that the observed activity may be attributed to the presence of phenolic and flavonoid compounds identified through qualitative LC–MS profiling. Given the exploratory nature of this study and the relatively high concentrations required to achieve bactericidal effects, further investigations are necessary to isolate active constituents, assess anti-biofilm properties, and evaluate safety and efficacy *in vivo* before considering potential therapeutic applications.

## Data Availability

The original contributions presented in the study are included in the article/supplementary material, further inquiries can be directed to the corresponding author.
